# Improving the production of 9α-hydroxy-4-androstene-3,17-dione from phytosterols by 3-ketosteroid-Δ^1^-dehydrogenase deletions and multiple genetic modifications in *Mycobacterium fortuitum*

**DOI:** 10.1186/s12934-023-02052-y

**Published:** 2023-03-16

**Authors:** Xiangcen Liu, Jingxian Zhang, Chenyang Yuan, Guilin Du, Suwan Han, Jiping Shi, Junsong Sun, Baoguo Zhang

**Affiliations:** 1 Lab of Biorefinery, Shanghai Advanced Research Institute, Chinese Academy of Sciences, No. 99 Haike Road, Pudong, Shanghai, 201210 China; 2 University of Chinese Academy of Sciences, Beijing, 100049 China; 3 School of Life Science and Technology, ShanghaiTech University, Shanghai, 201210 China

**Keywords:** 9α-hydroxy-4-androstene-3,17-dione, Phytosterol, 3-ketosteroid-∆^1^-dehydrogenase, *Mycobacterium fortuitum*

## Abstract

**Background:**

9α-hydroxyandrost-4-ene-3,17-dione (9-OHAD) is a significant intermediate for the synthesis of glucocorticoid drugs. However, in the process of phytosterol biotransformation to manufacture 9-OHAD, product degradation, and by-products restrict 9-OHAD output. In this study, to construct a stable and high-yield 9-OHAD producer, we investigated a combined strategy of blocking Δ^1^‑dehydrogenation and regulating metabolic flux.

**Results:**

Five 3-Ketosteroid-Δ^1^-dehydrogenases (KstD) were identified in *Mycobacterium fortuitum* ATCC 35855. KstD2 showed the highest catalytic activity on 3-ketosteroids, followed by KstD3, KstD1, KstD4, and KstD5, respectively. In particular, KstD2 had a much higher catalytic activity for C9 hydroxylated steroids than for C9 non-hydroxylated steroids, whereas KstD3 showed the opposite characteristics. The deletion of *kstDs* indicated that KstD2 and KstD3 were the main causes of 9-OHAD degradation. Compared with the wild type *M. fortuitum* ATCC 35855, MFΔ*kstD*, the five *kstDs* deficient strain, realized stable accumulation of 9-OHAD, and its yield increased by 42.57%. The knockout of *opccr* or the overexpression of *hsd4A* alone could not reduce the metabolic flux of the C22 pathway, while the overexpression of *hsd4A* based on the knockout of *opccr* in MFΔ*kstD* could remarkably reduce the contents of 9,21 ‑dihydroxy‑20‑methyl‑pregna‑4‑en‑3‑one (9-OHHP) by-products. The inactivation of FadE28-29 leads to a large accumulation of incomplete side-chain degradation products. Therefore, *hsd4A* and *fadE28-29* were co-expressed in MFΔ*kstD*Δ*opccr* successfully eliminating the two by-products. Compared with MFΔ*kstD*, the purity of 9-OHAD improved from 80.24 to 90.14%. Ultimately, 9‑OHAD production reached 12.21 g/L (83.74% molar yield) and the productivity of 9-OHAD was 0.0927 g/L/h from 20 g/L phytosterol.

**Conclusions:**

KstD2 and KstD3 are the main dehydrogenases that lead to 9-OHAD degradation. Hsd4A and Opccr are key enzymes regulating the metabolic flux of the C19- and C22-pathways. Overexpression of *fadE28-29* can reduce the accumulation of incomplete degradation products of the side chains. According to the above findings, the MF-FA5020 transformant was successfully constructed to rapidly and stably accumulate 9-OHAD from phytosterols. These results contribute to the understanding of the diversity and complexity of steroid catabolism regulation in actinobacteria and provide a theoretical basis for further optimizing industrial microbial catalysts.

**Supplementary Information:**

The online version contains supplementary material available at 10.1186/s12934-023-02052-y.

## Introduction

**Fig. 1:**
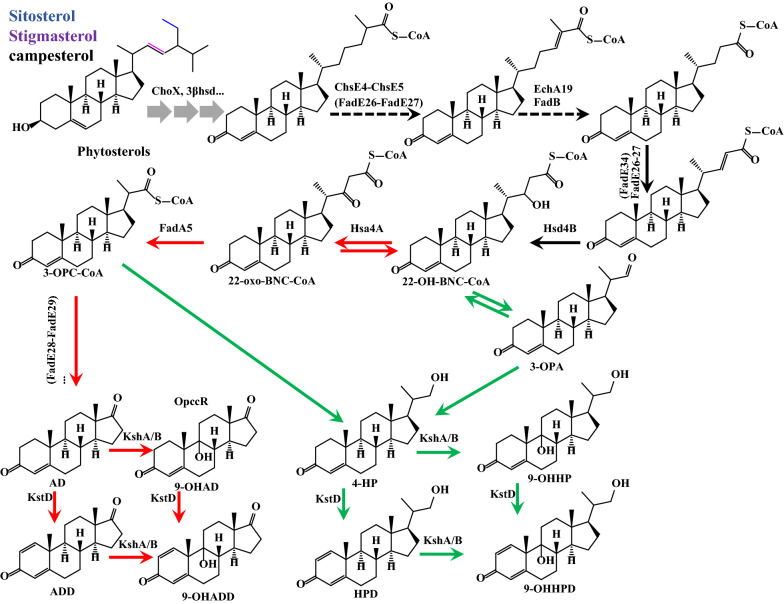
Schematic diagram of phytosterol metabolism in *Mycobacterium*. The red arrow represents the C19-pathway, the blue arrow represents the C22 pathway, and the dotted lines represents uncertain responses. *Chox* Cholesterol oxidase, *Hsd4A* 17β‑hydroxysteroid dehydrogenase/β‑hydroxyacyl‑CoA dehydrogenase, *FadA5* acetyl‑CoA acetyltransferase/thiolase, *ChsEs* acyl‑CoA dehydrogenases, *KstD* 3‑ketosteroid‑Δ^1^‑dehydrogenase, *KSH* 3‑ketosteroid‑9α‑hydroxylase, *AD* 4-androstene-3,17-dione, *ADD* 1,4-androstadiene-3,17-dione, *9-OHAD* 9α-hydroxy-4-androsten-3,17-dione, *4-HP* 21-hydroxy-20-methyl-pregna-4-en-3-one; *HPD* 21-hydroxy-20-methyl-pregna-1,4-diene-3-one, *9‑OHHP* 9,21‑dihydroxy‑20‑methyl‑pregna‑4‑en‑3‑one, *3-OPC-CoA* 3-oxo-4-pregnene-20-carboxyl-CoA. *3-OPA* 3-oxo-4-pregnene-20-carboxyl aldehyde.

Steroid drugs, the second largest classification of drugs after antibiotics, play a key role in disease prevention and clinical application [1, 2]. Synthesis of steroid precursors by phytosterol biotransformation has attracted considerable attention owing to resource constraints and environmental pressures [1, 3, 4]. Biochemical and genomic studies have shown that microorganisms participate in phytosterol degradation via similar metabolic pathways [5, 6]. The interruption of these metabolic pathways often leads to the accumulation of steroid intermediates [7], such as 9α-hydroxyandrost-4-ene-3,17-dione (9-OHAD) and 9,21-dihydroxy-20-methyl-pregna-4-en-3-one (9-OHHP) (Fig. 1), which are the key products of the C19 and C22 metabolic branches in steroid degradation.

9-OHAD, an important precursor in the manufacture of glucocorticoid drugs [8], can be obtained from steroids via two routes of microbial transformation. One route is that the hydroxyl is directly introduced into the C9 position of 4-androstene-3,17-dione (AD) by microorganisms with a hydroxylation function[9, 10]. However, the low activity of 3-ketosteroid 9α-hydroxylase (KSH), the hydrophobicity of AD, and its toxic effects on cells restrict the high-concentration conversion of AD [4]. Another form of biotransformation that directly converts phytosterols into 9-OHAD has become an area of interest owing to its cost-effective manufacture. However, low conversion rates and accumulation of by-products due to the hydrophobicity of sterols and the complexity of microbial metabolic pathways limit industrial production. For example, in addition to 9-OHAD, by-products such as AD, 9-OHHP, and 9,24-dihydroxychol-4-en-3-one (9,24-DHC) accumulate in *Mycobacterium* sp. 2–4 M and *Mycobacterium* sp. VKM Ac-1817D [11, 12]. The mutants of *M. neoaurum* ATCC 25795 led to the accumulation of 7.33 g/L 9-OHAD from 15 g/L phytosterols, while the conversion rate of phytosterols was only 67% [13]. The *M. neoaurum* HGMS2 mutants could convert 80 g/L phytosterol into 37.2 g/L 9-OHAD, but the molar yield was only 40.3% [14].

The integrity of 9-OHAD is highly susceptible to 3-Ketosteroid-Δ^1^-dehydrogenases (KstD) activity. KstD can remove hydrogen atoms of C-1 and C-2 in the ring A of 9-OHAD and form 9α-hydroxyandrost-1,4-diene-3,17-dione (9-OHADD). 9-OHADD undergoes a spontaneous ring B opening reaction owing to structural instability and then degraded [15, 16]. This means that any residual KstD activity could lead to the degradation of 9-OHAD. However, redundancy and the broad-spectrum catalytic substrate of KstD increase the difficulty in developing 9-OHAD producers [11, 17–20]. For example, the transcription level of KstD1 was significantly upregulated by phytosterols in *M. neoaurum* DSM 1381, but KstD2 exhibited the highest dehydrogenation activity on AD [18, 21]. In *Arthrobacter simplex* CGMCC 14539, KstD3 and KstD5 exhibited strong organic solvent tolerance and a clear preference for 4-ene-3-oxosteroids, but KstD4 had the broadest substrate profile [17]. The diversity of KstDs confers the ability for strains to utilize different steroid substrates while raising the difficulty in developing 9-OHAD producers.

Accumulation of many by-products with similar structures usually occurs in steroid producers. For example, small amounts of the C22 intermediates, 9-OHHP and 9,24-DHC, also accumulate during the conversion of phytosterols to 9-OHAD by *Mycobacterium *sp. VKM Ac-1817D [22]. A similar phenomenon also occurred in *Mycobacterium *sp. LY-1, and *M. neoaurum* ATCC 25795 [23, 24]. These undesired by-products not only hinder the purification and refinement process of 9-OHAD but also reduce its yield. The accumulation of C22 by-products, such as 9-OHHP and 21-hydroxy-20-methyl-pregna-4-en-3-one (4-HP), indicated that the side chain cleavage process was unexpectedly interrupted [11, 25]. The 17-hydroxysteroid/22-OH-BNC-CoA dehydrogenase (Hsd4A), a key enzyme located at the bifurcation of phytosterol metabolism pathway. Overexpression or knockout of *hsd4A* can regulate the accumulation of C19 or C22 products [26]. Peng et al. reported that a dual-role reductase mnOpccr (designated Opccr in this study), in the phytosterol catabolism, which engages in two different metabolic branches to produce the key intermediate 4-HP through a 4-e reduction of 3-oxo-4-pregnene-20-carboxyl-CoA (3-OPC-CoA) and 2-e reduction of 3-oxo-4-pregnene-20-carboxyl aldehyde (3-OPA), respectively [27] (Fig. 1). Considering the competitiveness of the C19 and C22-pathway, the inhibition of C22 metabolic flux could reduce C22 by-products and increase 9-OHAD production. Therefore, *hsd4A* and *Opccr* are important targets for genetic modification to develop microorganisms capable of converting phytosterols into 9-OHAD without C22 by-products.

*Mycobacterium fortuitum* ATCC 35855 can rapidly convert phytosterols to 9-OHAD, and likewise, suffers from the coexistence of multiple by-products and product degradation [15, 28], which indicates that the KstD and C22-pathway are active in phytosterol biotransformation. However, the mechanism of 9-OHAD degradation and by-product production in *M. fortuitum* ATCC 35855 remains unclear. In this study, the roles of different KstD homologs in *M. fortuitum* ATCC 35855 were identified and analyzed, and a 9-OHAD producer was obtained by the knockout of all the KstDs. Additionally, the production of 9-OHAD was further enhanced by blocking the C22 pathway through the regulation of multiple key genes. Here, we studied the residual *kstD*s and the genes related to the accumulation of by-products for the first time. Our findings add to the understanding of the complexity and diversity associated with steroid catabolism regulation in *M. fortuitum* and establish a theoretical foundation for the optimization of industrial microbial biocatalysts.

## Results

### In silico analysis of the putative KstDs

Using whole-genome sequencing, five putative *kstD*s (gene0972, gene0864, gene1922, gene4300, and gene4305) were identified and designated as *kstD1*, *kstD2*, *kstD3*, *kstD4*, and *kstD5*. The physicochemical properties of the five KstDs are displayed in Additional file 1: Table S2. All KstDs were hydrophilic, and no distinct signal was predicted in their leading region. As shown in Additional file 1: Fig. S1, the five isoenzymes are flavoproteins containing a consensus N-terminal flavin adenine dinucleotide (FAD)-dependent domain, GSG(A/G)(A/G)(A/G)X17E [20]. Four residues, considered significant for flavoprotein functioning, were found to be highly conserved in the five KstDs: Tyr119, Tyr487, and Gly491, in the FAD-binding domain, and Tyr318 in the catalytic domain [29, 30].

**Fig. 2:**
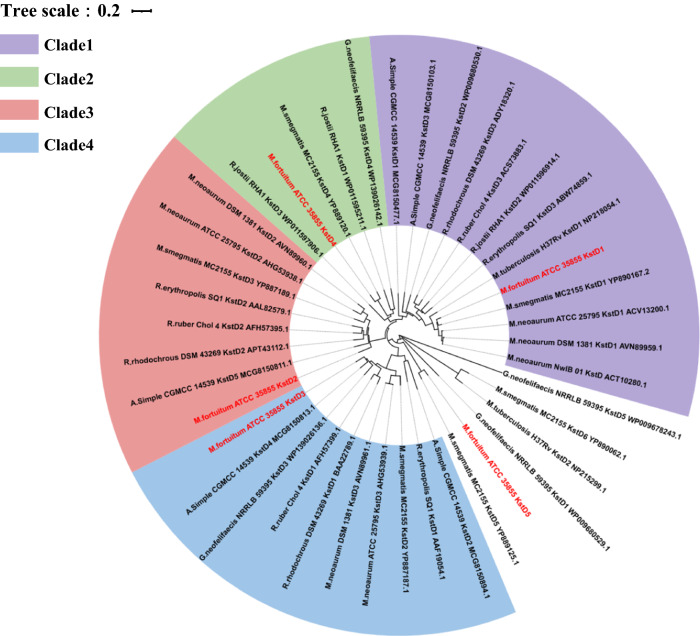
Phylogenetic analysis of the putative 3-Ketosteroid-Δ1-dehydrogenases (KstDs) in *Mycobacterium fortuitum* ATCC 35855 and some representative orthologues. KstDs in *M. fortuitum* ATCC 35855 is marked in red. Different colors refer to different evolutionary branches. The scale length is set at 0.2

Several KstD isozymes which the physicochemical properties and the roles in steroid metabolism have been studied were selected for phylogenetic analysis. In the phylogenetic tree (Fig. 2), these KstD isozymes were divided into four clusters, where KstD1, KstD4, KstD2, and KstD3 from *M. fortuitum* ATCC 35855 were located in clade 1–4, separately. The KstD in the same clade exhibited similar physicochemical properties. In clade1, KstD3 from *R. erythropolis* SQ1 [31], KstD1 from *M. tuberculosis* H37Rv [21], KstD1 in *M. neoaurum* ATCC 25795[23], and KstD3 from *R. ruber* Chol-4 [32] showed a narrow substrate range and active on 5α-3-ketosteroids. In addition, KstD3 from *R. rhodochrous* DSM 43269 [33] did not play any role in AD conversion to 9-OHAD. Although some KstDs belong to clade 1, there are differences between different small branches. For example, KstD1 from *M. neoarum* ATCC 25795 participated in the conversion of 9-OHAD, and KstD1 from *M. neoaurum* 1381 [30] and KstD3 from *A. simplex* CGMCC 14539 [34] had obvious preference for 4-ene-3-oxosteroids. In clade3, KstD2 from *R. erythropolis* SQ1, KstD2 from *M. neoaurum* 1381, KstD2 from *R. rhodochrous* DSM, and KstD2 from *R. ruber* Chol-4 all have high 4-ene-3-oxosteroids dehydrogenation activity and broad substrate preference. Most of these KstDs play a leading role in the process of AD conversion to ADD. In particular, KstD5 from *A. simplex* CGMCC 14539 also has strong resistance to organic solvents and good activity on substrates with or without substituents at C11 position. In clade3, KsdD1 from *R. ruber* Chol-4 and KstD3 from *M. neaurum* DSM 1381 were proved not to participate in AD conversion. Similar to other microorganisms, the five KSTD isozymes of *M. fortuitum* ATCC 35855 are distributed in different branches, which may reflect that the functionally diverse of five KstDs and the ability of *M. fortuitum* ATCC 35855 to use a variety of steroid substrates.

**Fig. 3:**
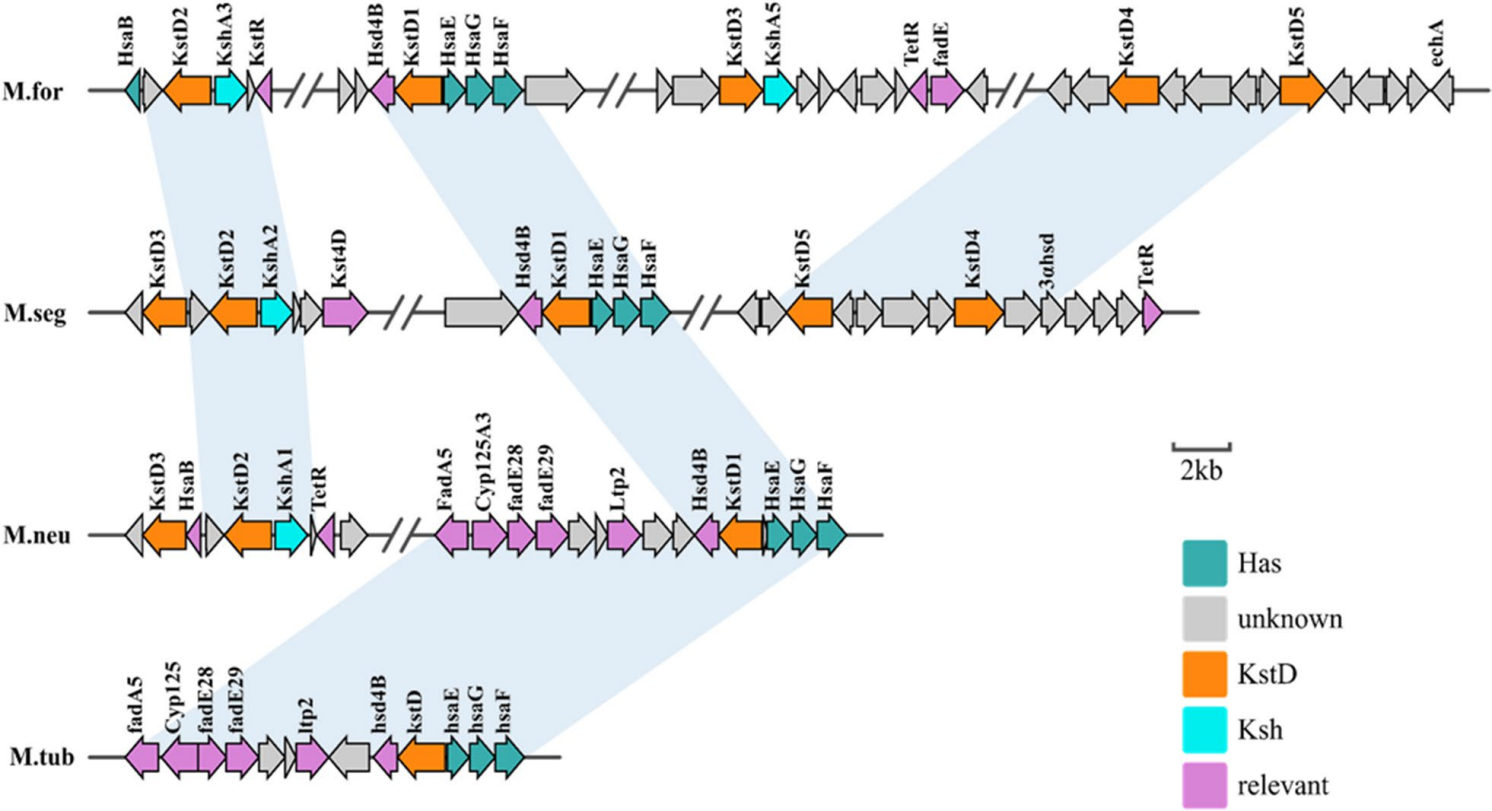
Schematic of the genomic organization of *kstD* homologs in *M. fortuitum* ATCC 35855 and other mycobacteria. M.for, *Mycobacterium fortuitum* ATCC 35855; M.neo, *M. neoaurum* DSM 1381; M.tub, *M. tuberculosis* H37Rv, M.seg, *M. smegmatis* mc^2^155. The direction and size of genes are indicated as an arrow according to the scale at the bottom. Orthologous counterparts are included in light blue. KstD encodes putative 3-ketosteroid-Δ^1^-dehydrogenase; Ksh encodes putative terminal oxygenase of KSH; HsaE-G encodes putative hydratase, 4-hydroxy-2-oxovalerate aldolase, and aldehyde dehydrogenase; Relevant indicates other related enzymes involved in the steroid metabolism process

Insights into their genomic allocation that distinguished the status of various *kstD*s homologs are shown in Fig. 3. Five *kstD*s were found in the four steroid degradation gene clusters. The loci of *kstD2* and *kstD3* were distant in the characterized steroid catabolism gene cluster, and each of them accompanied by a *kshA* was organized in a neighboring community on the genome, which suggested that KstD2, KstD3, and KshA probably played a vital role in the metabolism of the steroid nucleus [23]. *kstD1* is surrounded by *hsaE*, *hsaG*, *hsaF,* and *hsd4B*, and a similar structure can be found in *M. neoaurum* DSM 1381, *M. smegmatis* mc^2^155, and *M. tuberculosis* H37Rv. However, *kstD4* and *kstD5* werelocated in one gene cluster, and no sterol degradation-related genes with well-defined functionality were found in their vicinity. Moreover, only one highly conserved orthologous counterpart, *kstD4-5* has been found in *M. smegmatis* mc^2^155. This indicates that KstD4 and KstD5 are substitutable in steroid metabolic engineering.

### Heterologous expression of KstD homologs

The expression of KstDs in *Escherichia coli* BL21 (DE3) was identified by sodium dodecyl-sulfate polyacrylamide gel electrophoresis (SDS-PAGE) (Additional file 1: Fig. S3). The enzyme activities of KstDs in *E. coli* BL21 (DE3) were investigated using the crude cell-free extracts and listed in Table 1. Although all KstDs could catalyze the Δ^1^-dehydrogenation of steroids, such as AD, 9-OHAD, HP, and 9-OHHP, some significant differences were observed in their substrate preferences. The low catalytic activities of KstD4 and KstD5 implied their negligible role in steroid metabolism. However, KstD1 showed higher activity for AD, 9-OHAD, 4-HP, and 9-OHHP, with activities of 2.92, 3.43, 3.17, and 2.75 mU/mg, respectively. KstD2 displayed the highest specific activity toward four substrates: 261.75, 533.41, 2104.38, and 1203.52 mU/mg, respectively, and KstD3 displayed a weak dehydrogenation catalytic activity: 60.61, 51.78, 19.03 and 13.77 mU/mg, respectively. The specific enzyme activity of KstD2 and KstD3 for substrates was more than 10 folds higher than that of KstD1, KstD4, and KstD5, which indicated that the two KstDs may play a major role in the degradation of 9-OHAD. The specific enzyme activity of KstD2 to 4-HP was twice that of AD. In contrast, compared to the substrate 4-HP, KstD3 showed higher specific activity for AD. For C9 hydroxylated steroids, the specific activity of KstD2 and KstD3 to 9-OHAD was 1.5 folds higher than that of 9-OHHP. KstD2 had higher specific enzyme activity for C9 hydroxylated steroids than C9 non-hydroxylated steroids as a substrate, while KstD3 had the opposite effect. The physicochemical properties of the two enzymes were consistent with those of KstDs from other strains, such as *Mycobacterium *sp. VKMAc-1817D [12], *M. fortuitum* ATCC 6842 and its mutant HA-1 [28], and some *Rhodococcus* [13, 34]. The distinct substrate preferences demonstrated that these KstD homologs may play diverse roles in the sterol catabolic pathway.

**Table 1:** The KstD enzyme activity (mU/mg total soluble protoplast protein) of ATCC 35855

Substrate	KstD1		KstD2		KstD3		KstD4		KstD5	
	Specific Activity^a^	Rel. Activity^b^	Specific Activity	Rel. Activity	Specific Activity	Rel. Activity	Specific Activity	Rel. Activity	Specific Activity	Rel. Activity
AD	2.92 ± 1.14	100	261.75 ± 4.51	100	60.61 ± 1.43	100	0.93 ± 0.14	100	0.95 ± 0.18	100
4-HP	3.43 ± 1.06	117.37	533.41 ± 2.60	203.78	51.78 ± 2.87	85.43	0.55 ± 0.14	59.93	0.83 ± 0.32	88.03
9-OHAD	3.17 ± 0.80	108.46	2104.38 ± 43.3	803.94	19.03 ± 1.43	31.41	0.29 ± 0	31.56	0.29 ± 0.49	30.89
9-OHHP	2.75 ± 0	94.09	1203.52 ± 43.3	459.78	13.77 ± 2.48	22.73	1.35 ± 0.25	145.15	1.16 ± 0.18	122.14

^a^Values (shown as mean ± S.E.M., n = 3) simply indicate the relative performance on substrates using the identical KstD enzyme

^b^The activity of each KstD is initially evaluated using AD as the substrate. This is set at 100% to indicate the relative activity against other substrates. The inhibition effect of methanol and PMS on the KstD activity was ignored

### Assessment of *kstD* transcription

**Fig. 4:**
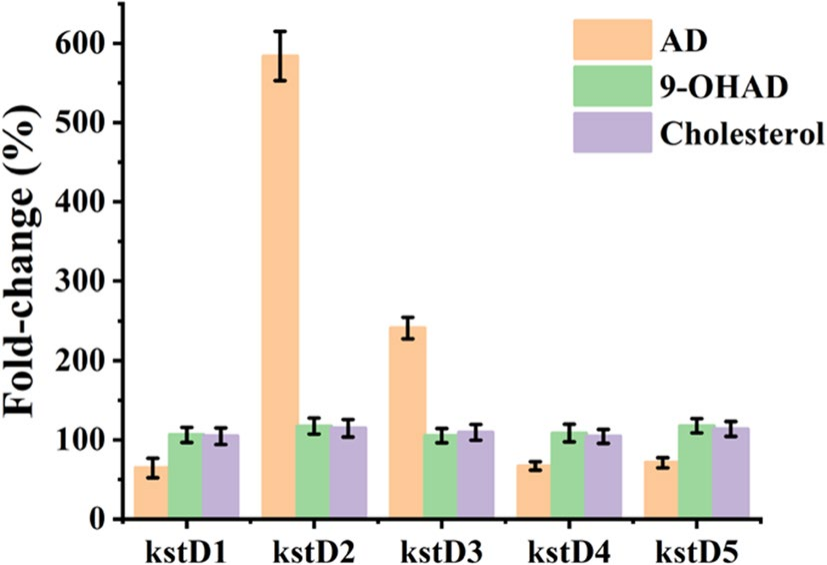
The transcription of *kstD* genes in *M. fortuitum* ATCC 35855. The fold-change indicates the ratios of mRNA levels in *M. fortuitum* ATCC 35855 growing on steroid inducers relative to glycerol

To understand the expression of *kstD*s in vivo, transcriptome analysis of ATCC 35855 induced by cholesterol, AD, or 9-OHAD was performed. As shown in Fig. 4, the transcription levels of *kstD2* and *kstD3* increased by 5.74 and 2.46-folds when induced with AD. However, the transcription levels of *kstD1*, *kstD4,* and *kstD5* decreased. Although 9-OHAD was the optimum substrate for *kstD2*, it did not cause a significant increase at the transcriptional level. Similarly, all *kstD*s were not significantly upregulated under cholesterol treatment, and the transcription levels increased by less than 50%. This performance of *kstD*s was significantly different from that of other homologous analogs in steroid-metabolizing strains such as *M. neoaurum* DSM1381 and *M. neoaurum* ATCC 25,795.

### Improving the 9-OHAD accumulation by knocking out kstDs

**Fig. 5:**
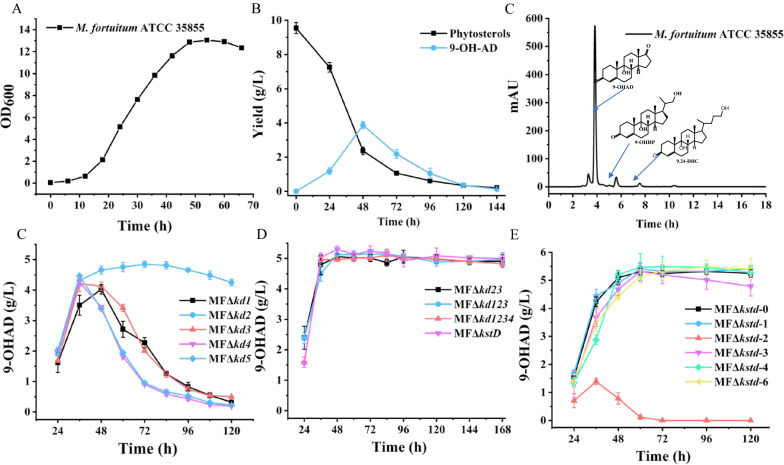
Phenotypic analyses of *M. fortuitum* ATCC 35855 and mutants. **A** Growth curve of *M. fortuitum* ATCC 35855; **B** time course of phytosterols degradation and 9-OHAD accumulation in *M. fortuitum* ATCC 35855; **C** HPLC of phytosterol degradation at 48 h by *M. fortuitum* ATCC 35855; **D**–**F** 9-OHAD accumulation of single or multiple *kstD*s-deleted mutants and single *kstD* complemented strains in the medium MF/01 supplemented with 10 g/l phytosterol. Standard deviations of the biological replicates are represented by error bars

Compared with other steroid transformation strains [14, 36, 37], *M. fortuitum* ATCC 35855 has the advantages of a short growth cycle and fast degradation of phytosterols (Fig. 5AB). To elucidate the roles of KstDs in 9-OHAD degradation, single and multiple deletions of *kstD* mutants were constructed. Phytosterol transformation was performed with these mutants to determine the function of the five KstDs in the degradation of 9-OHAD. Within 72 h of fermentation, 10 g/L of phytosterols was completely consumed by all mutants. The yield of 9-OHAD reached the maximum after about 48 h of inoculation with each of the mutants. As shown in Fig. 5D, MFΔ*kd2* remarkably produced 9-OHAD to a maximum of 4.94 g/L at 72 h, but a slower degradation occurred and decreased to 4.23 g/L after 120 h. The fastest degradation occurred in MFΔ*kd4* and MFΔ*kd5* after 48 h, which showed the same phenomenon as *M. fortuitum* ATCC 35,855, and all of them reached the peak of 9-OHAD yield (about 3.9 g/L) at 48 h, and then decreased rapidly, which suggested that KstD2 plays the major role in the A-ring dehydrogenation of 9-OHAD combined with enzyme activity data. Compared with *M. fortuitum* ATCC 35855, MFΔ*kd1* and MFΔ*kd3* showed a weak improvement in the degradation of 9-OHAD in the middle of fermentation, which indicated that KstD1 and KstD3 also contribute to the degradation of 9-OHAD, although their dehydrogenation activity is not high. Considering that the other four KstD homologs also displayed dehydrogenase activity, they were successively knocked out in MFΔ*kd2*. In Fig. 5E, all the different multiple *kstD* deficient mutants showed that the characteristics of 9-OHAD were not degraded even if the fermentation time was extended to 168 h. The maximum yield of 9-OHAD of MFΔ*kd23* was 5.05 g/L, and MFΔ*kd123,* MFΔ*kd1234,* and MFΔ*kstD* accumulated 5.14, 5.18 and 5.29 g/L 9-OHAD, respectively. The five *kstDs* deletion mutant, MFΔ*kstD*, seemed to be superior with the extension of fermentation time (Fig. 5E). A single *kstD* compensation was performed in MFΔ*kstD* to further verify the performance of each KstD in the accumulation of 9-OHAD from phytosterols (Fig. 5F). The yield of 9-OHAD reached a maximum of 5.19 g/L, and 9-OHAD began to decrease slightly at 72 h with MFΔ*kstD-3*. MFΔ*kstD-2* only accumulated 1.4 g/L 9-OHAD, and it was completely degraded within 72 h. However, the accumulation of 9-OHAD did not show a significant decrease in MFΔ*kstD-1*, MFΔ*kstD-4*, and MFΔ*kstD-5*.

### Elimination of 9-OHHP by-products by regulating the metabolic flux of C19 and C22 pathways

**Fig. 6:**
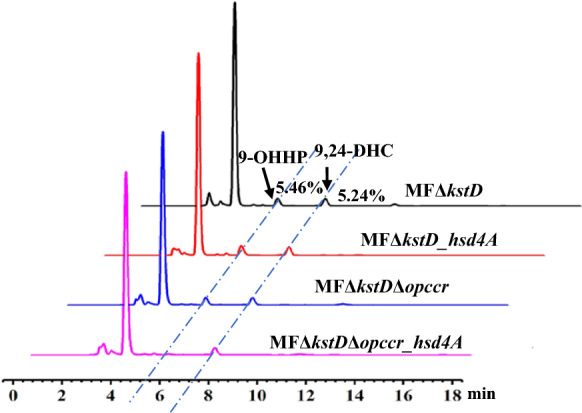
Elimination of by-products 9,21‑dihydroxy‑20‑methyl‑pregna‑4‑en‑3‑one (9-OHHP) in conversion of phytosterols of mutants

Compared with *M. fortuitum* ATCC 35855, the output of 9-OHAD increased from 3.87 to 5.29 g/L in MFΔ*KstD*. However, the purity of 9-OHAD was only 80.24% owing to the existence of by-products such as 9-OHHP (5.46%) and 9,24-DHC (5.24%) (Fig. 6). The C22 intermediate 9-OHHP indicated that the C22 steroidal metabolic pathway may play a weak role in cholesterol metabolism (Fig. 1). Knockout or overexpression of Hsd4A, could manipulate the phytosterols metabolic flux to accumulate either C19 or C22 steroid products. However, overexpression of exogenous *hsd4A* in MFΔ*kstD* had no noticeable effects on the reduction of the accumulation of 9-OHHP (Fig. 6). A dual-role reductase, mnOpccr, can convert 3-OPA-CoA and 3-OPA into 4-HP in *M. neoaurum* CCTCC AB2019054 [22]. Therefore, we speculated whether there is an enzyme with the same function of mnOpccr in *M. fortuitum* ATCC 35855 that contributes to the accumulation of 4-HP or 9-OHHP. Subsequently, *opccr*, the homologous gene (76.3% identity) of *mnOpccr* was found in the *M. fortuitum* ATCC 35855 genome by BLAST and then knocked out in MFΔ*kstD*. Compared with MFΔ*kstD*, the ratio of 9-OHHP in the product did not change significantly with MFΔ*kstD*Δ*opccr* (Fig. 6). Overexpression of *hsd4A* or knockout of *opccr* alone could not effectively reduce the accumulation of 9-OHHP. However, MFΔ*kstD*Δ*opccr*_*hsd4A*, in which the exogenous hsd4A was overexpressed in MFΔ*kstD*Δ*opccr*, significantly reduced the accumulation of the by-product 9-OHAD. The proportion of 9-OHHP in the product was only 0.89%, compared with MFΔkstD, and decreased by 83.70%. In addition, knockdown of the side-chain degradation genes suggested that overexpression of fadE28-29 could reduce the accumulation of the by-product 9,26-DHC (Additional file 1: Fig. S4), and the proportion of 9,24-DHC in the product decreased from 5.24 to 2.04%。

Based on the above results, MF-FA5020, a mutant in which *hsd4A* and *fadE28-29* were co-expressed in MFΔ*kstD*Δ*opccr*, was constructed to attempt to remove the by-products, 9-OHHP and 9,24-DHC, simultaneously. As shown in Additional file 1: Fig. S4, the proportions of 9-OHHP and 9,24-DHC in the product were reduced to less than 3%, and the proportion of 9-OHAD was 90.14%.

### Evaluation of the 9-OHAD producer

**Fig. 7:**
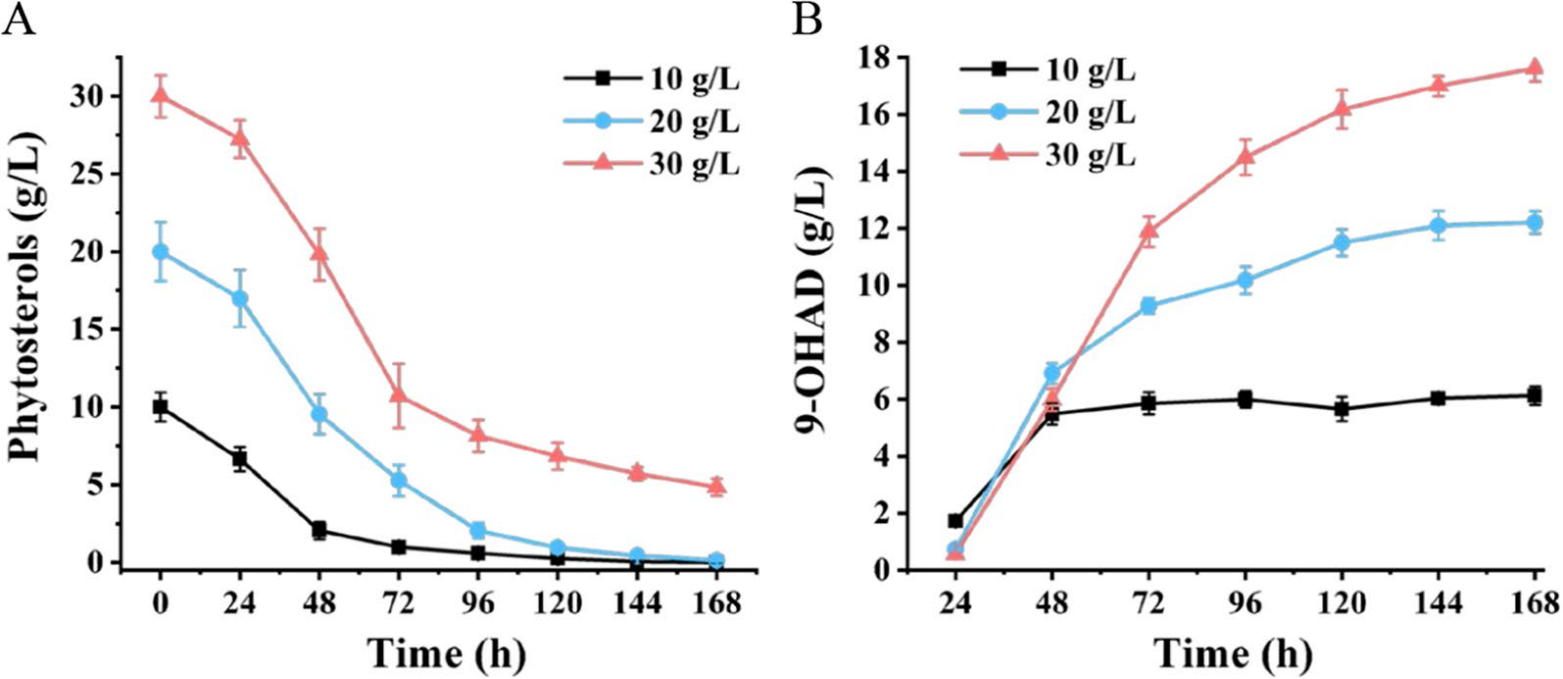
Time profiles of 9α-hydroxyandrost-4-ene-3,17-dione (9-OHAD) accumulation by MF-FA5020 with different concentrations of phytosterols. **A** The concentration of remained phytosterols; **B** real-time yield of 9-OHAD. Standard deviations of the biological replicates are represented by error bars

To evaluate the ability of MF-FA5020 to transform phytosterols into 9-OHAD, higher concentrations of phytosterols were incubated with the mutants. Figure 7 illustrates the conversion rates of 10, 20, and 30 g/L of phytosterols, respectively, subjected to 168 h of fermentation. Specifically, the mutant could completely convert 10 g/L phytosterols into 6.12 g/L 9-OHAD within 96 h, and the molar yield of 9-OHAD was 84.18%. Compared with *M. fortuitum* ATCC 35855, the yield of 9-OHAD from MF-FA5020 increased by 53.85%. 12.21 g/L 9-OHAD accumulated within 144 h from 20 g/L phytosterols, and the molar yield of 9-OHAD was 83.68%. Furthermore, phytosterols were also completely consumed. However, even if the fermentation process was prolonged, phytosterols were not exhausted when they increased to 30 g/L, wherein 17.62 g/L 9-OHAD was produced. The molar yield of 9-OHAD was 80.53%, and the molar conversion of phytosterol was 84.46%.

## Discussion

9-OHAD is an important precursor for the preparation of β-methadone, dexamethasone, and other steroid drugs. *Mycobacterium fortuitum* ATCC 35855 is an excellent candidate producer of 9-OHAD owing to its high conversion of phytosterols. However, the residual KstDs and C22-pathway lead to 9-OHAD degradation and by-product accumulation, which hinders industrial application. In this study, the residual *kstD*s and the genes related to the accumulation of by-products were studied for the first time.

Five possible *kstD* genes were identified in *M. fortuitum* ATCC 35855. The heterologous expression and transcriptional response of *kstD*s indicated that KstD2 exhibited the highest activity for all substrates, followed by KstD3, and the other three kstDs showed negligible activity for 3-ketosteroids. The results implied that KstD2 and KstD3 are the main contributors to the dehydrogenation reaction in steroid metabolism. KstD2 and KstD3 showed different preferences for C9 hydroxylated and non-hydroxylated steroids, respectively, suggesting that KstD2 may be mainly involved in the 9-OHAD pathway, while KstD3 plays a role in the AD pathway. This result is consistent with the previous conclusion that *M. fortuitum* has two different Δ^1^-dehydrogenase activities [28]. Although KstD1 from *M. fortuitum* ATCC 35855 had highly identical aa sequence with KstD1 from *M. neoaurum* DSM 1381 and *M. neoaurum* ATCC 25795 (92% and 88%, respectively), high dehydrogenase activity towards AD was not detected with KstD1 in *M. fortuitum* ATCC 35855, and the simultaneous knockout or overexpression of KstD1 had no effect on 9-OHAD. This may be caused by mutations in the non-conserved sites of KstD. For example, the S138L mutation decreased the activity of KstD1 in *M. neoaurum* [35].

MFΔ*kd2* accumulated the maximum amount of 9-OHAD, but degraded significantly after 72 h, which indicated the non-negligible effect of other dehydrogenases. Different combinations of *kstDs* deletion mutants, in which *kstD2* and *kstD3* were deleted simultaneously, achieved a stable accumulation of 9-OHAD. This proved that these two KstDs are mainly responsible for the degradation of 9-OHAD in *M. fortuitum* ATCC 35855. Most of the potential 9-OHAD producers reported failed to obtain ideal 9-OHAD production due to residual KstD activity [14, 23, 36]. For example, although KstD1 provides 99% dehydrogenation capacity in *R. erythropolis* SQ1, only when KstD1 and KstD2 are inactivated simultaneously can 9-OHAD be accumulated. Additionally, kstD3 has no catalytic capacity for 9-OHAD but has high dehydrogenation activity for the saturated A-ring and produces a 1,4-diene structure [13, 20, 32, 37]. Similarly, KstDs with diverse functions have been found in many steroid producers, such as *M. neoaurum* ATCC25795 [23], *M. neoaurum* DSM1381 [30], and *M. smegmatis* [31, 33]. Therefore, the inactivation of all KstDs is a necessary condition for developing promising 9-OHAD biocatalysts, although the specific function of low-activity KstD in sterol metabolism is unclear.

AD and 4-HP were not detected, indicating that the KSH hydroxylation activity in MFΔ*kstd* was sufficient; therefore, *M. fortuitum* ATCC 35855 is a potential host for producing C9 hydroxysteroid intermediates. However, in addition to the main product, 9-OHAD, the proportion of the two by-products, 9-OHHP and 9,24-DHC, is 11%. The two by-products are accumulated owing to the incomplete degradation of the side chains. However, in this study, neither the overexpression of *hsd4a* nor the inactivation of *opccr* reduced the metabolic flux of the C22-pathway and lowered the proportion of 9-OHHP. These results suggest that it is not feasible to modify the C19-pathway or C22-pathway alone. On the one hand, Hsd4A is a bidirectional functional enzyme and its catalytic direction is affected by the redox level of coenzyme (NADH/NAD +) [26]; so even if the activity of Hsd4A was increased, the metabolic flux to the 9-OHHP pathway was unchanged. On the other hand, the catalytic properties of Opccr may favor non-hydroxylated steroids, and the contribution of Opccr to 9-OHHP is not as substantial compared to 4-HP, because C9 hydroxylation occurs prior to other catalytic reactions in *M. fortuitum* ATCC 35855. Based on these results, simultaneous inactivation of *opccr* and overexpression of *hsd4A* in MFΔ*kstD* reduced the by-product 9-OHHP to less than 1%. The complexity of the side chain degradation reaction, characterized by another by-product, 9.24-DHC, which was reduced by overexpressing *fadE28-29*, requires further study to delineate its specific underlying mechanism.

The production of 9-OHAD did not increase with an increase in phytosterol concentration at the early stage of transformation. This phenomenon was probably caused by the hydrophobicity of sterols, insufficient oxygen content, and the inhibition of high concentrations of sterols on the growth of bacteria. The low solubility of phytosterols greatly limits the uptake efficiency of sterol molecules by microorganisms. Although many methods can increase the water solubility of sterols, they also produce deleterious effects. For instance, *M. neoaurum* HGMS2 used soybean oil to increase solubility during the fermentation of high-concentration phytosterols, and the conversion rate of phytosterols was more than 90%. However, the yield of 9-OHAD was 40.3 g/L, and the molar yield was only 66.8% [14, 38]. The nonionic surfactant TX-40 has been proven to promote phytosterol biotransformation, but it increases production costs. Furthermore, resting cell fermentation is not suitable for large-scale industrial applications [38–40]. However, in this study, MF-FA5020 was constructed by combining the inactivation of KstDs and blocking the C22 pathway, which yielded 12.21 g/L 9-OHAD from 20 g/L phytosterols. The conversion rate of phytosterol was 95%, and the molar yield and maximum productivity of 9-OHAD were 83.74% and 0.0927 g/L/h, respectively. The purity of 9-OHAD in the product was 90.12%. Compared to the existing 9-OHAD producers, MF-FA5020 has incomparable advantages for industrialization in terms of phytosterol conversion, product purity and yield, and fermentation processes.

## Conclusions

During the phytosterol conversion of *M. fortuitum* ATCC 35855, KstD2 was the major contributor to 9-OHAD degradation, while the function of KstD3 was secondary, and the dehydrogenation activity of the other three KstDs was not indispensable. The removal of the five KstDs successfully prevented the degradation of 9-OHAD. Moreover, the deletion of the key gene, *opccr*, and the overexpression of *hsd4A*, successfully reduced the metabolic flux of the C22 pathway and blocked the accumulation of the by-product 9-OHHP. Additionally, FadE28-29 complemented the activity of related enzymes during side-chain degradation. By using the combined strategy of gene inactivation and augmentation, the production of 9-OHAD was successfully maximized, and the by-products were reduced, which serves as a promising tool to overcome the current limitations associated with the production of clinically relevant steroid intermediates.

## Materials and methods

### Bacterial strains, plasmids, reagents, and culture conditions

*Mycobacterium fortuitum *subsp.* fortuitum* ATCC 35855 was purchased from the American Type Culture Collection (ATCC), and the other strains used in this study are listed in Table 2 and Additional file 1: Table S1. LBT medium (10.0 g/L NaCl, 10.0 g/L peptone, 5.0 g/L yeast extract, and 2.0 g/L Tween-80 (pH 7.0)) was used to aerobically cultivate *M. fortuitum* at 30 °C, 200 rpm. Transcriptome studies of steroid degradation genes were carried out in LBT medium with 5 g/L cholesterol, 9-OHAD, AD, or glycerol (blank control). The MF/01 medium that consists of 15 g/L corn steep powder, 10 g/L glucose, 6 g/L NaNO_3_, 0.7 g/L (NH_4_)_2_HPO_3_ and 2.0 g/L Tween 80 (pH 7.5) was used for phytosterol biotransformation. 60 g/L phytosterol mother liquor [16]: mix phytosterol and HP-β-CD in water according to the ratio of 1:3(m/m), stirred 15 min, sonicated 20 min, repeat three times. For the different concentrations fermentation of phytosterol, an appropriate amount of well-mixed phytosterol mother liquor is taken and diluted with MF/01 medium to the desired concentration.

**Table 2:** Strains used in this study

Strains	Description	Source
*E. coli*		
BL21-*kd1/kd2/kd3/kd4/kd5*	Recombinant BL21 (DE3) cells, possessing pET28a-*kstD1/kstD2/kstD3/kstD4/ kstD5*	This study
*Mycobacterium fortuitum*		
ATCC 35855	9-OH-AD as a main product during sterol consumption and degradation with increasing fermentation time	ATCC
MFΔ*kd1/2/3/4/5*	*kstD1/2/3/4/5* deleted mutant of ATCC 35855	This study
MFΔ*kd*23	*kstD2&3* deleted mutant of ATCC 35855	This study
MFΔ*kd*123	*kstD1&2&3* deleted mutant of ATCC 35855	This study
MFΔ*kd*1234	*kstD1&2&3&4* deleted mutant of ATCC 35855	This study
MFΔ*kstD*	*kstD1&2&3&4&5* deleted mutant of ATCC 35855	This study
MFΔ*kstD*-0	MFΔ*kstD* carrying a vacant p40	This study
MFΔ*kstD*-1/2/3/4/5	*kstD1/2/3/4/5* over-expression in MFΔ*kstD* via p40-*kstD1/2/3/4/5*	This study
MFΔ*kstD*_*hsd4A*	*hsd4A* from *M. neoaurum* DSM 44074 over-expression in MFΔ*kstD* via p40-*Hsd4A*	This study
MFΔ*kstD*Δ*opccr*	*opccr* deleted mutant of MFΔ*kstD*	This study
MFΔ*kstD*Δ*opcc*r_*hsd4A*	*hsd4A* from *M. neoaurum* DSM 44074 over-expression in MFΔ*kstD*Δ*opccr* via p40-*Hsd4A*	This study
MFΔ*kstD*Δ*opcc*rΔ*fadE26-27*	*fadE26-27* deleted mutant of MFΔ*kstD*Δ*opcc*r	This study
MFΔ*kstD*Δ*opcc*r Δ*fadE28-29*	*fadE28-29* deleted mutant of MFΔ*kstD*Δ*opcc*r	This study
MFΔ*kstD*Δ*opcc*r_* fadE28-29*	*fadE28-29* from over-expression in MFΔ*kstD*Δ*opccr* via p40-60	This study
MF-FA5020	*hsd4A* and *fadE28-29* from over-expression in MFΔ*kstD*Δ*opccr* via p40-60	This study

*Escherichia coli* DH5α and BL21 (DE3), carrying recombinant plasmids, were used for cloning and protein expression, respectively. 0.1 mM of IPTG was added to induce the expression of KstDs. Furthermore, 50 μg/mL kanamycin, 50 μg/mL apramycin, 200 μg/mL 5-bromo-4-chloro-3-indolyl-β-d-galactopyranoside (X-gal), and 20 g/L sucrose were supplemented into the medium for the selection of *E. coli* and *Mycobacterium* transformants when necessary.

Phytosterols were purchased from Yunnan Biological Products Co., Ltd. (Yunnan, China); AD, ADD, 4-HP, HPD, 9-OHHP, and 9-OHAD were purchased from Sigma-Aldrich (Shanghai, China); 2,6-dichlorophenolindophenol (DCPIP) and phenazine methosulfate (PMS) were purchased from Shanghai Titan Science & Technology Co., Ltd. All other reagents used were of analytical grade or higher unless noted otherwise.

### Bioinformatic analysis

The whole genome and transcriptome of *M. fortuitum* ATCC 35,855 were sequenced by Shanghai Majorbio Co., Ltd. The DNA or mRNA was isolated, sequenced, and analyzed as described by Zhang [22]. The phylogenetic tree was constructed using MEGA 11 software with ClustalW and the neighbor-joining algorithm. The ProtParam and ProtScale tools on the webserver (http://expasy.org) were used to putatively analyze the physico-chemical properties and hydrophobicity/hydrophilicity of each KstD homolog. TMHMM (http://www.cbs.dtu.dk/services/TMHMM-2.0/) and SignalP5.0 (http://www.cbs.dtu.dk/services/SignalP/) were used to predict the transmembrane region and signal peptides of each KstD. Multiple sequence alignments were performed using MEGA 11 and espript3.0 (https://espript.ibcp.fr/ESPript/cgi-bin/ESPript.cgi). Bioedit and Vector NTI programs were used to perform local-blast alignments within the genome data.

### Determination of kstD enzyme activity

The ORF of five KstDs in *M. fortuitum* ATCC 35855 were amplified using pairs of primers (28k1-f&R, 28k2-f&R, 28k3-f&R, 28k4-f&R, and 28k5-f&R, Additional file 1: Table S1). The PCR products were cloned into a *HindIII*/*BamHI*-digested pET-28a ( +) vector and then verified by PCR and DNA sequencing. Subsequently, recombinant plasmids were introduced into *E. coli* BL21 (DE3) cells for enzymatic studies. A final concentration of 0.1 mM IPTG induction was used for induction when the culture after inoculation grew to OD_600_ = 0.5. After 12 h at 20 °C, the cells were harvested by centrifugation (8000 ×*g*, 5 min) from 50 mL cultures and washed twice with 50 mL Tris–HCl buffer (50 mM Tris–HCl, pH 7.5), and subsequently resuspended in the same buffer. The cells were sonicated for 10 min in an ice-water bath. Afterward, the supernatant of free cell extracts (12,000 × *g*, 4 °C, 10 min) was used for enzyme activity assays with different substrates against the blank control of empty pET-28a ( +), and the expression of KstDs in *E. coli* BL21 (DE3) was confirmed by SDS-PAGE.

The enzyme activities of crude enzyme solutions of five KstDs were measured spectrophotometrically at 600 nm (ε600 = 18.7 × 10^3^ m^−1^ M^−1^) using a NanoDrop2000 (Thermo Scientific, Waltham, MA, USA) at 30 °C [17]. 2,6-dichlorophenolindophenol (DCPIP) and phenazine methosulfate (PMS) were used as electron acceptors. The reaction mixture consisted of 50 mM Tris–HCl pH 7.0, 0.12 mM DCPIP, 1.5 mM PMS, 500 μM steroid substrates in methanol (2%) [13], and crude enzyme solution. The Bradford method was employed to determine the total protein content in the supernatants of the cell-free extracts and cultures. One unit of enzyme activity is defined as the reduction of 1 µmol DCPIP/min. Specific activities were defined as micromoles per milligram per minute (U/mg/min).

### Deletion of *kstDs* homologs and other related genes

Gene deletion was performed using the methods described by Parish and Stoker [18]. To simplify the knockout plasmid construction steps, the plasmids p2NIL and pGoal19, were combined into one knockout suicide plasmid (Additional file 1: Fig.S2) and the detailed steps are as follows. Primers p2nIL-F/R were used to eliminate the redundant fragment between the *PstI* and *SalI* digestion sites in the p2nIL plasmid and introduce a new *AlfII* digest site, and the p2nIL-1 plasmid was formed by circular PCR. The screening marker gene fragments, *ScaB* and *LacZ,* from the pGoal19 plasmid, were amplified using p2n3-F/R primers and then inserted between the *PacI* and *EcoRI* digestion sites of the p2nIL-1 plasmid to obtain pDCO-1. To avoid microbial contamination as well as to improve the efficiency of recombination screening, a second resistance gene fragment, apramycin (Seq. 1 in Additional file 1), was synthesized by GENEWIZ and inserted into the *SphI* digest site of the pDCO-1 plasmid, thus resulting in the suicide delivery plasmid, pKADel, which was used to perform unmarked deletions in *M. fortuitum* ATCC 35855.

For the initial deletion of *kstD1*, two fragments approximately 1.2 kb upstream and downstream, flanking the ORF of the target *kstD1* gene, were cloned with primers, *kstD*1-U-f/r and *kstD1*-D-f/r, respectively, after which they were ligated into an approximately 2.5 kb fragment by overlapping PCR based on the designed primers, *kstD1*-U-f and *kstD1*-D-r, respectively. Subsequently, the 2.5 kb fragment was cloned into the digestion site between *AlfII* and *SalI* of pKADel, and the knockout plasmid pKADel-*kstD1* was constructed. The plasmid was transferred into mycobacterial cells using electroporation as described by Parish and Stoker [27]. After the two-step selection process, the unmarked deletion mutants of *kstD1* were selected and confirmed by PCR using the *kstD1*-U-f and *kstD1*-D-r primers. The correct *kstD*1 mutant strains were determined by gene sequencing to confirm that the *kstD1* gene was successfully deleted. The knockout method for other genes was performed as previously described for *kstD1* and the corresponding primers are listed in Additional file 1: Table S1. The double deletion of *kstD3* and *kstD*2 was performed via the abrogation of *kstD3* in *kstD2* deficiency strains with pKADel-*kstD3* and other multiple gene knockout bacteria were constructed using similar methods.

For the deletion of *fadE26-27, fadE28-29,* or *opccr*, plasmids pKADel-*fadE26-27,* pKADel-*fadE28-29 and* pKADel-*opccr* were constructed with pairs of primers *fadE26-27*-U-f/r and *fadE26-27*-D-f/r, *fadE28-29*-U-f/r, and *fadE28-29*-D-f/r, and *opccr*-U-f/r and *opccr*-D-f/r, respectively (Additional file 1: Table S1). Then, *fadE26-27*, *fadE28-29,* or *opccr* knockout strains were obtained and confirmed by PCR and sequencing.

### Complementation and overexpression of genes

The constitutive plasmid, pMV306, was employed as an expression vector to complement the expression of steroid degradation genes in mutants. Since pMV306 does not have a promoter, plasmid p40 was constructed by inserting the promoter, psmyc [19], between the *XbaI*/*EcoRI* digestion sites of pMV306. The fragments of *kstDs* were amplified from *M. fortuitum* ATCC 35855 using the corresponding primers, p40k*-F&R (Additional file 1: Table S1). *Hsd4A* and *fadE28-29* fragments were amplified from *M. neoaurium* DSM 44074 with hsd4a-F/R and fade28-29-F/R. Afterward, all gene fragments were inserted between the *AflII* and *HindIII* digestion sites of plasmid p40 to create five *kstDs* complement plasmids p40-*k1/k2/k3/k4/k5* and expression plasmids p40-*hsd4A* and p40-*fadE28-29*, respectively (Additional file 1: Table S1). For the co-expression of *hsd4A* and *fadE28-29* in mutants, primers 4afad-F/R (containing a Shine-Dalgarno sequence for ribosome binding) were used to amplify the *fadE28-29* fragment from p40-*fadE28-29*. This new *fadE28-29* fragment was inserted into the *AflII/SalI* site of plasmid p40-*Hsd4A* to create the co-expression plasmids for *hsd4A* and *fadE28-29*, and p40-*hsd4A&fadE28-29*.

### Analytical methods

The bioconversion of steroids was monitored for 5–7 days and sampled every 12 h, and three replicate experiments were performed for each strain. The samples of the culture (0.5 ml) were extracted on a vortex mixer for 5 min with 1 ml ethyl acetate before centrifugation at 12,000 ×*g* for 2 min. The supernatant organic phase of the sample was analyzed using thin-layer chromatography (TLC) and high-performance liquid chromatography (HPLC). TLC plates (HSGF254 2.5 × 5 cm) were used as a rapid qualitative approach to detect the steroid bioconversion products and residual substrate with ethyl acetate-hexane (6: 4) as a developing solvent. For HPLC, the supernatant organic phase of the sample was re-dissolved in methanol after drying and then filtered through a 0.22 μm microporous membrane. HPLC (Agilent Technologies, Santa Clara, CA, U.S.A.) with an Agilent Extend-C18 column (5 μm, 4.6 × 250 mm, 40 °C) was used to determine the types of different steroid compounds. For the substrates AD, 4-HP, 9-OHAD, 9-OHHP, and 9.24-DHC, methanol/water (80: 20, v/v) was used as the mobile phase at a flow rate of 0.8 mL/min and ultraviolet detection at 254 nm was employed.

### Accession numbers

The genome sequencing information of *M. fortuitum* ATCC 35855 has been deposited in the GenBank database with accession number CP110127. The accession numbers of *kstD1*, *kstD2*, *kstD3*, *kstD4* and *kstD5* gene sequences from *M. fortuitum* ATCC 35855 are OP729262.1, OP729263.1, OP729264.1, OP729265.1, and OP729266.1, respectively. The accession numbers of *hsd4A*, *opccr*, *fadE26-27* and *fadE28-29* gene sequences are OP729274.1, OP729275.1, OP729276.1-OP729277.1, and OP729278.1-OP729279.1, respectively.

## Supplementary Information

**Additional file 1: Fig S1.** Amino acid sequence alignment of known KstDs. **Fig. S2. **Construction process of suicide plasmids pKADel. **Fig. S3.** SDS-PAGE analyses of soluble KstDs in cell-free extracts of recombinant *E. coli* cells. **Fig. S4. **Elimination of by-products 9,24-DHC in conversion of phytosterols of mutants. **Table S1.** Primers, plasmids, and strains used in this study. **Table S2.** Summary of bioinformatics analysis and secondary structure prediction of the putative KstDs.

## Data Availability

All data generated and analyzed during this study are included in this published article and its additional files.

## Abbreviations

AD: 4-androstene-3,17-dione
AD: 1,4-androstadiene-3,17-dione
9-OHAD: 9α-hydroxy-4-androsten-3,17-dione
4-HP: 21-hydroxy-20-methyl-pregna-4-en-3-one
HPD: 21‑dihydroxy‑20‑methyl‑pregna-1,4-diene-3-one
9-OHHP: 9,21‑dihydroxy‑20‑methyl‑pregna‑4‑en‑3‑one
9,24-DHC: 9,24-dihydroxychol-4-en-3-one
KstD: 3-ketosteroid-∆^1^-dehydrogenase
KSH: 3‑ketosteroid‑9α‑hydroxylase
Hsd4A: 17β‑hydroxysteroid dehydrogenase/β‑hydroxy acyl‑CoA dehydrogenase
FadA5: Acetyl‑CoA acetyltransferase/thiolase
