# Supplementary material for: Improving the production of 9α-hydroxy-4-androstene-3,17-dione from phytosterols by 3-ketosteroid-Δ1-dehydrogenase deletions and multiple genetic modifications in Mycobacterium fortuitum
Source: Microb Cell Fact. 2023 Mar 16;22:53. doi: 10.1186/s12934-023-02052-y (PMC10018825; doi:10.1186/s12934-023-02052-y)
Supplement: Supplementary file 1 — Additional file 1: Fig S1. Amino acid sequence alignment of known KstDs. Fig. S2. Construction process of suicide plasmids pKADel. Fig. S3. SDS-PAGE analyses of soluble KstDs in cell-free extracts of recombinant E. coli cells. Fig. S4. Elimination of by-products 9,24-DHC in conversion of phytosterols of mutants. Table S1. Primers, plasmids, and strains used in this study. Table S2. Summary of bioinformatics analysis and secondary structure prediction of the putative KstDs. [file 12934_2023_2052_MOESM1_ESM.docx]

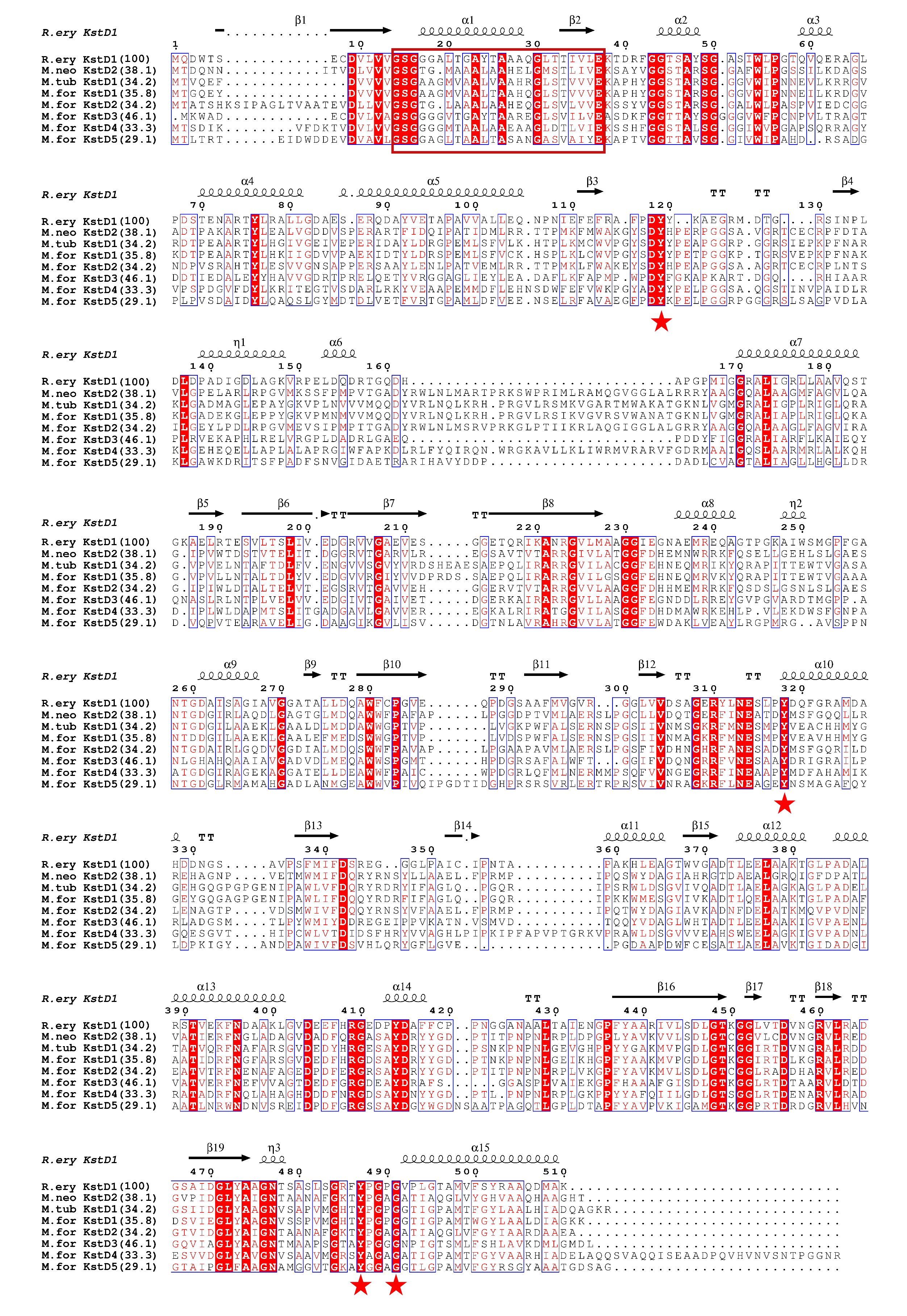


**Fig. S1. Amino acid sequence alignment of known KstDs.** The FAD-binding domain is boxed in red. Line1 indicates the protein structure of KstD1 of *R. erythropolis* SQ1 and active site residues and residues involved in coordination of a FAD in KsdD1 from *R. erythropolis* SQ1 are indicated by red asterisks. R. ery, *R. erythropolis* SQ1; M. neo, M. neoaurum DSM 1381; M. tub, *M. tuberculosis* H37Rv; M.for, *Mycobacterium fortuitum subsp. fortuitum* ATCC 35855. The number in parentheses represents the aa identity (%) between KsdDs of this strain and KsdD1 of *R. erythropolis* SQ1


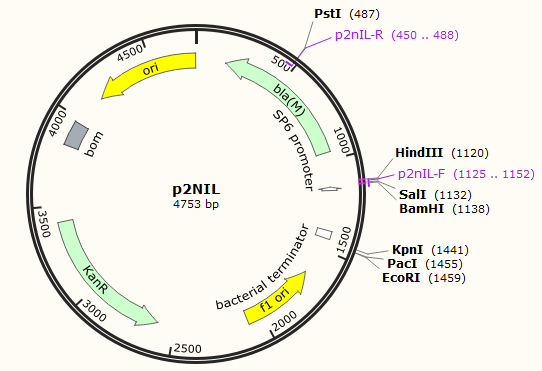

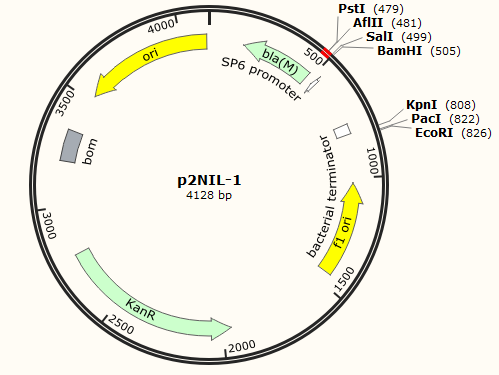

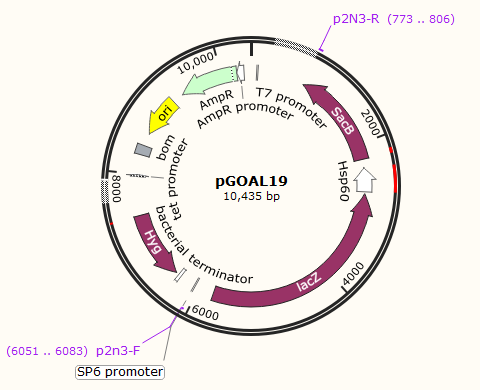

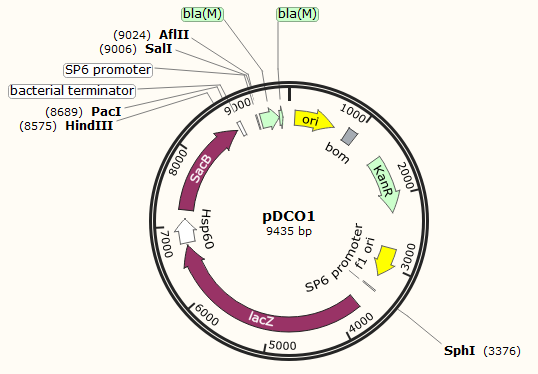

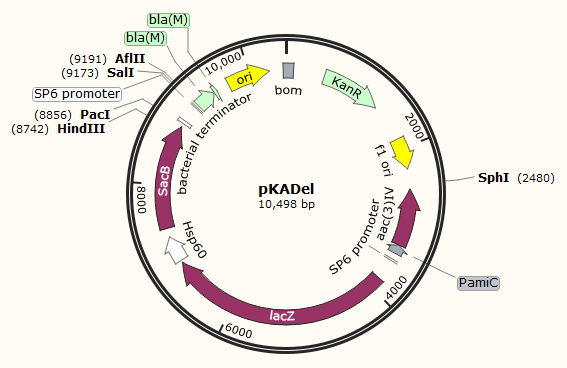

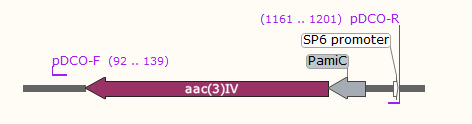


**Fig. S2 Construction process of suicide plasmids pKADel.**

**Seq. 1 Pamic-apramycin Sequence**:

aacaggtgagattacggagaacggggcttgtggccgtccctgtcgtgtcgtaacgtgtccacaacgttgcagttcactgcagtcatgagcgcccggcgagtcactaaggagggatccgtgcaatacgaatggcgaaaagccgagctcatcggtcagcttctcaaccttggggttacccccggcggtgtgctgctggtccacagctccttccgtagcgtccggcccctcgaagatgggccacttggactgatcgaggccctgcgtgctgcgctgggtccgggagggacgctcgtcatgccctcgtggtcaggtctggacgacgagccgttcgatcctgccacgtcgcccgttacaccggaccttggagttgtctctgacacattctggcgcctgccaaatgtaaagcgcagcgcccatccatttgcctttgcggcagcggggccacaggcagagcagatcatctctgatccattgcccctgccacctcactcgcctgcaagcccggtcgcccgtgtccatgaactcgatgggcaggtacttctcctcggcgtgggacacgatgccaacacgacgctgcatcttgccgagttgatggcaaaggttccctatggggtgccgagacactgcaccattcttcaggatggcaagttggtacgcgtcgattatctcgagaatgaccactgctgtgagcgctttgccttggcggacaggtggctcaaggagaagagccttcagaaggaaggtccagtcggtcatgcctttgctcggttgatccgctcccgcgacattgtggcgacagccctgggtcaactgggccgagatccgttgatcttcctgcatccgccagaggcgggatgcgaagaatgcgatgccgctcgccagtcgattggctga

* The red characters represent *Pamic* promoters and black characters represent apramycin gene sequences.


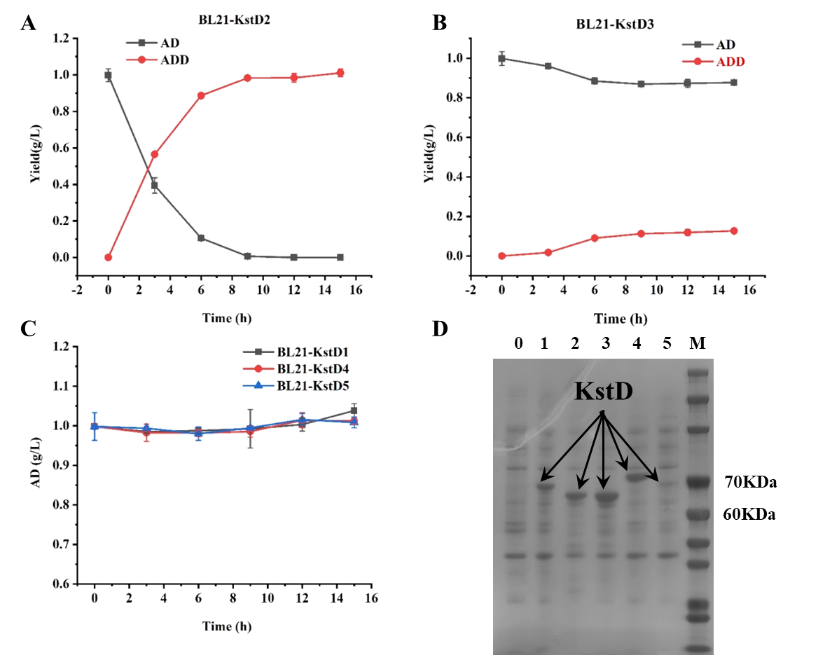


**Fig. S3 Heterologous expression of KstD and transformation of recombinant cells.**

A, Transformation of AD by KstD2 recombinant cells; B, Transformation of AD by KstD3 recombinant cells; C, Transformation of AD by KstD1、KstD4 or KstD5 recombinant cells;D, SDS-PAGE analyses of soluble kstDs in cell-free extracts of recombinant *E. coli* cells. M, protein Maker; 0, *E. coli* BL21 harboring vacant pET-28a; 1, KstD1 expression of BL21-*kd1*; 2, KstD2 expression of BL21-*kd2*; 3, KstD3 expression of BL21-*kd3*; 4, KstD4 expression of BL21-*kd4*; 5, KstD5 expression of BL21-*kd5*.

**Elimination of by-products 9-OHHP by overexpression of *fadE28-29***


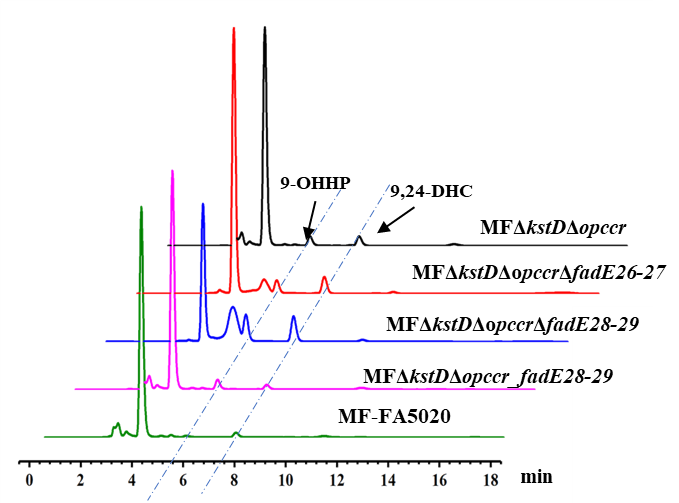


**Fig. S4 Elimination of by-products 9,24-DHC in conversion of phytosterols of mutants.**

The accumulation of 9,24-DHC, an intermediate in the incomplete degradation of side chain, may be caused by insufficient activities of side chain degradation enzymes. Therefore, its accumulation can be reduced by increasing the activity of side chain degradation enzymes theoretically. Some genes related to side chain degradation, such as *fadE26-27*, *fadE28-29*, *fadE34*, etc., were knocked out in MFΔ*kstD* to verify the possible functions in the process of generating 9,24-DHC. Surprisingly, a significant yield change of 9,24-DHC was found in *fadE26-27* and *fadE28-29* deletion mutants, separately. As showed in Fig.6B, the proportion of 9,24-DHC increased from 5.24% to 9.78% after *fadE26-27* was deleted, while increased to 16.79% after *fadE28-29* was knocked out. Since *fadE28-29* has more significant contribution to the accumulation of product 9,24-DHC than *fadE26-27*, *fadE28-29* was overexpressed in MFΔ*kstD*Δ*opccr* to try to eliminate the accumulation of by-product 9,24-DHC. It is satisfactory that the proportion of 9,24-DHC in the product decreased from 5.24% to 2.04%, and the purity of 9-OHAD in the product increased from 78.6% to 85.12%.

**Table S1 Primers, plasmids, and strains used in this study.**

| Name | Description | Source/reference |
| --- | --- | --- |
| Plasmids |  |  |
| DH5α | *E. coli* expression vector, *Kan^R^* | Novagen |
| pET-28a(+) | *E. coli* expression vector, *Kan^R^* | Novagen |
| pET-28a-*kstD1/2/3/4/5* | Expression plasmid pET28a possessing orf of *kstD1/2/3/4/5* | This study |
| p2NIL | Plasmid for allelic exchange, non-replicative in mycobacterium, Kan^R^ | Parish and Stoke (2000) |
| pGoal19 | Hyg^R^ Pag_85_-*lacZ* P_hsp60_-*sacB*, *PacI* cassette vector, Amp^R^ | Parish and Stoke (2000) |
| P2NIL-1 | Plasmid for allelic exchange, non-replicative in mycobacterium, Kan^R^ | This study |
| pDCO1 | Plasmid for allelic exchange, Pag_85_-*lacZ* P_hsp60_-*sacB*, Kan^R^ | This study |
| pKADel | Plasmid for allelic exchange, Pag_85_-*lacZ* P_hsp60_-*sacB*, Apr^R^, Kan^R^ | This study |
| pKADel-*kstD1/2/3/4/5* | pKADel carrying two homologous arms (i.e. upstream and downstream) of *kstD1/2/3/4/5* | This study |
| pKADel*-fadE26-27* | pKADel carrying two homologous arms of *fadE26-27* | This study |
| pKADel*-fadE28-29* | pKADel carrying two homologous arms of *fadE28-29* | This study |
| pKADel-*opccr* | pKADel carrying two homologous arms of *opccr* | This study |
| pMV306 | Integrative vector with single copy in *mycobacterium*, without the heat shock (hsp60) promoter, Kan^R^ | [1] |
| p40 | pMV306 with Psmyc promoter, Kan^R^ | This study |
| p40-*k1/k2/k3/k4/k5* | p40 possessing *kstD1/kstD2/kstD3/kstD4/ kstD5 genes* | This study |
| p40-*hsd4A* | p40 possessing *hsd4A* from *M. neoaurum* DSM 44074 | This study |
| p40-*fadE28-29* | p40 possessing*fadE28-29* from *M. neoaurum* DSM 44074 | This study |
| p40-*hsd4A&fadE28-29* | p40 possessing *hsd4A* and *fadE28-29* from *M. neoaurum* DSM 44074 | This study |
| Primers |  |  |
| *E. coli* expression | | |
| 28k1-F | gtggtggtggtggtgctcgagctactttttccctgcaatgtcgagtgc | |
| 28k1-R | ttaagaaggagatataccatggtgttctacatgactggacaggagtacgac | |
| 28k2-F | gtggtggtggtggtgctcgagctacgcttcggccgcgtcgcgag | |
| 28k2-R | actttaagaaggagatataccgtgaccgccaccagccacaagag | |
| 28k3-F | gtggtggtggtggtgctcgagtcacaagtccatccctaacatgtct | |
| 28k3-R | actttaagaaggagatataccatgaagtgggccgacgaatgtgat | |
| 28k4-F | gtggtggtggtggtgctcgagtcatcggtttcctcctggggtgttgctgac | |
| 28k4-R | actttaagaaggagatataccatgacctcagacatcaaggtgttcgac | |
| 28k5-F | gtggtggtggtggtgctcgagtcagcccgctgagtcgcccgt | |
| 28k5-R | actttaagaaggagatataccatgacattgacccgcaccgaaattgac | |
| PCR for deletion | | |
| p2nIL-F | cgttgttgccattgctgcagcttaagactagtcacgtggtcgacggatcctatgtattc | |
| p2nIL-R | tgactagtcttaagctgcagcaatggcaacaacgttgcgcaaactattaactg | |
| p2n3-F | gcgctgcccggattacaggggcatgcatttaggtgacactatagaatacataggatctg | |
| p2n3-R | attggtaccgcggccgcttaattaaatcggcattttcttttgcgtttttatttgttaac | |
| pDCO-F | tgcgctgcccggattacaggggcatgcgccatcatggccgcgggctag | |
| pDCO-R | ttctatagtgtcacctaaattcccggagcaaacgcaatcac | |
| *kstD*1-U-f | taggatccgtcgaccacgtggaccctcggattccgggtcgat | |
| *kstD*1-U-r | ctcgttgttcgggatccacaccccac | |
| *kstD*1-D-f | ggggtgtggatcccgaacaacgagcgcaccgatctgaagggccg | |
| *kstD*1-D-r | cgttgttgccattgctgcagagaagcggctggtacgtgcgc | |
| *kstD*2-U-f | taggatccgtcgaccacgtgccattgaacgctcgtctggttccaactg | |
| *kstD*2-U-r | accgggataggttttgccgaagcccgtgccggaaccgac | |
| *kstD*2-D-f | ttcggcaaaacctatcccggtgctacatcgccgctcgcgac | |
| *kstD*2-D-r | cgttgttgccattgctgcagggacatcggtttcactgttcagggacaagac | |
| *kstD*3-U-f | taggatccgtcgaccacgtggaccatcgtcgggatcaacccgac | |
| *kstD*3-U-r | atcggatttccgccgcccggggaccccgcgaccaggacatcacat | |
| *kstD*3-D-f | ccgggcggcggaaatccgat | |
| *kstD*3-D-r | cgttgttgccattgctgcagacacgatcacctgcgagcgg | |
| *kstD*4-U-f | tatagaatacataggatccgtcgacccgcgttacgaccagggcgc | |
| *kstD*4-U-r | tgtgcgactgactgttgtgcggcggccaatgcagctgtcatac | |
| *kstD*4-D-f | tgacagctgcattggccgccgcacaacagtcagtcgcacaacagatt | |
| *kstD*4-D-r | cgttgttgccattgctgcaggcccgggtgacctcggcg | |
| *kstD*5-U-f | tatagaatacataggatccgtcgacgtcggccatcagccatttgatccgggc | |
| *kstD*5-U-r | ccggcgccgccgtagcccccgctgcccagtacggcgac | |
| *kstD*5-D-f | gggcagcgggggctacggcggcgccggcggaac | |
| *kstD*5-D-r | cgttgttgccattgctgcagcgagcatcctcgccgcgacaccac | |
| *opccr*-U-f | tatagaatacataggatccccgaccacgatcgcgatggcctgcag | |
| *opccr* -U-r | ggacaggatccgggtgatcacg | |
| *opccr* -D-f | atcacccggatcctgtccgtccgcagcacgcgaagcctgc | |
| *opccr* -D-r | cgttgttgccattgctgcagccgtggccggtcgacgagcggt | |
| fadE26-27-U-f | tatagaatacataggatccgcgttgagcgaccctccacgagactg | |
| fadE26-27-U-r | gccctcggacgagctgagcgc | |
| fadE26-27-D-f | cgctcagctcgtccgagggcctggccgccaagcagacggagttc | |
| fadE26-27-D-r | cgttgttgccattgctgcagccgtgccgacccgcacataggacg | |
| fadE28-29-U-f | tatagaatacataggatccggctggctacgcgcacggcttg | |
| fadE28-29-U-r | cagtgcatcccaagtgttgtcgcgctc | |
| fadE28-29-D-f | acttgggatgcactgaccaagcgcaacctcgtgatcaccttc | |
| fadE28-29-D-r | cgttgttgccattgctgcagcgatgctcactgtgctcccatggtcaacg | |
| *Mycobacterium fortuitum* expression | | |
| p40k1-F | ccatcaggaggaatcctgcgtgttctacatgactggacaggagtacgacg | |
| p40k1-R | gtgcgaagtgattcctccgcttaagctactttttccctgcaatgtcgagtgcg | |
| p40k2-F | ccatcaggaggaatcctgcgtgaccgccaccagccacaagagcat | |
| p40k2-R | gtgcgaagtgattcctccgcttaagctacgcttcggccgcgtcgcgag | |
| p40k3-F | ccatcaggaggaatcctgcatgaagtgggccgacgaatgtgat | |
| p40k3-R | gtgcgaagtgattcctccgcttaagtcacaagtccatccctaacatgtctttcac | |
| p40k4-F | ccatcaggaggaatcctgcatgacctcagacatcaaggtgttcgacaaaaccg | |
| p40k4-R | gtgcgaagtgattcctccgcttaagtcatcggtttcctcctggggtgttgct | |
| p40k5-F | ccatcaggaggaatcctgcatgacattgacccgcaccgaaattgact | |
| p40k5-R | gtgcgaagtgattcctccgcttaagtcagcccgctgagtcgcccgttgc | |
| hsd4a-F | ccatcaggaggaatcctgcatgaacgacaacccgatcgac | |
| hsd4a-R | gtgcgaagtgattcctccgcttaagtcaagaacccatgagctcagttgcg | |
| fade28-29-F | ccatcaggaggaatcctgcgtggacttcacgccgaagcccgaacag | |
| fade28-29-R | gcgaagtgattcctccgcttaagtcaccgggtcaccctcggcacct | |
| 4aFad-F | gctcatgggttcttgacggtaccatcaggaggaatc | |
| 4aFad-R | Ctagttaactacgtcgaccttaagtcaccgggtcaccctcggcac | |

**Table S2 Summary of bioinformatics analysis and secondary structure prediction of the putative KsdDs**

|  | KstD1 | KstD2 | KstD3 | KstD4 | KstD5 |
| --- | --- | --- | --- | --- | --- |
| Molecular formula | C_2721_H_4242_N_752_  O_795_S_20_ | C_2674_H_4174_N_746_  O_809_S_19_ | C_2432_H_3768_N_678_  O_743_S_14_ | C_2903_H_4484_N_812_  O_841_S_18_ | C_2497_H_3877_N_701_  O_776_S_13_ |
| Number of amino acid | 562 | 567 | 514 | 598 | 543 |
| Predicting protein size (KDa) | 60.851 | 60.326 | 54.841 | 64.79 | 56.55 |
| GC content (%) | 65.96 | 65.14 | 67.50 | 66.33 | 67.93 |
| Isoelectric point (PI) | 7.71 | 5.28 | 4.80 | 5.79 | 4.73 |
| Positively charged residue  Arg+Lys | 62 | 48 | 47 | 58 | 44 |
| Negatively charged  residue Asp+Glu | 61 | 62 | 71 | 69 | 67 |
| Stability | stable | stable | stable | stable | stable |
| Hydrophilic/  hydrophobic | Hydrophilic | Hydrophilic | Hydrophilic | Hydrophilic | Hydrophilic |
| Cell localization | membrane | membrane | membrane | membrane | membrane |
| Signal peptides | absent | absent | absent | absent | absent |
| Transmembrane region | exist | exist | exist | exist | exist |
| Second structure | | | | | |
| α-helix | 34.16 | 34.04 | 33.27 | 32.61 | 31.12 |
| β-turn | 8.01 | 6.70 | 7.39 | 7.19 | 8.47 |
| Random coil | 42.53 | 45.15 | 45.14 | 45.48 | 44.01 |
| Extended strand | 15.30 | 14.11 | 14.20 | 14.72 | 16.39 |

1. Stover CK, Delacruz VF, Fuerst TR, Burlein JE, Benson LA, Bennett LT, Bansal GP, Young JF, Lee MH, Hatfull GF, et al: **New Use of Bcg for Recombinant Vaccines.** *Nature* 1991, **351:**456-460.
